# Comparability of Pulmonary Nodule Size Measurements among Different Scanners and Protocols: Should Diameter Be Favorized over Volume?

**DOI:** 10.3390/diagnostics13040631

**Published:** 2023-02-08

**Authors:** Colin F. Gross, Lisa Jungblut, Sebastian Schindera, Michael Messerli, Valentin Fretz, Thomas Frauenfelder, Katharina Martini

**Affiliations:** 1 Diagnostic and Interventional Radiology, University Hospital Zurich, 8091 Zurich, Switzerland; 2 Faculty of Medicine, University of Zurich, 8006 Zurich, Switzerland; 3 Institute of Radiology, Cantonal Hospital of Aarau, 5001 Aarau, Switzerland; 4 Nuclear Medicine, University Hospital Zurich, 8091 Zurich, Switzerland; 5 Division for Radiology and Nuclear Medicine, Cantonal Hospital Winterthur, 8400 Winterthur, Switzerland

**Keywords:** screening, radiation dosage, pulmonary nodules, measurement, size

## Abstract

Background: To assess the impact of the lung cancer screening protocol recommended by the European Society of Thoracic Imaging (ESTI) on nodule diameter, volume, and density throughout different computed tomography (CT) scanners. Methods: An anthropomorphic chest phantom containing fourteen different-sized (range 3–12 mm) and CT-attenuated (100 HU, −630 HU and −800 HU, termed as solid, GG1 and GG2) pulmonary nodules was imaged on five CT scanners with institute-specific standard protocols (P_S_) and the lung cancer screening protocol recommended by ESTI (ESTI protocol, P_E_). Images were reconstructed with filtered back projection (FBP) and iterative reconstruction (REC). Image noise, nodule density and size (diameter/volume) were measured. Absolute percentage errors (APEs) of measurements were calculated. Results: Using P_E_, dosage variance between different scanners tended to decrease compared to P_S_, and the mean differences were statistically insignificant (*p* = 0.48). P_S_ and P_E(REC)_ showed significantly less image noise than P_E(FBP)_ (*p* < 0.001). The smallest size measurement errors were noted with volumetric measurements in P_E(REC)_ and highest with diametric measurements in P_E(FBP)_. Volume performed better than diameter measurements in solid and GG1 nodules (*p* < 0.001). However, in GG2 nodules, this could not be observed (*p* = 0.20). Regarding nodule density, REC values were more consistent throughout different scanners and protocols. Conclusion: Considering radiation dose, image noise, nodule size, and density measurements, we fully endorse the ESTI screening protocol including the use of REC. For size measurements, volume should be preferred over diameter.

## 1. Introduction

Late-stage lung cancers are considered to be one of the most lethal human cancers challenging current modern medicine [1,2]. According to the National Cancer Institute (Bethesda, MD, USA), the U.S. national expenditures for lung cancer care in the year 2020 were estimated at USD 23.8 billion [3], cementing it as one of the costliest cancers to date. Prior studies have shown that the successful early detection of lung cancers using low-dose computed tomography (CT) screening programs can reduce mortality rates substantially [4,5,6], partly due to the higher chance of curative treatment in early-stage lung cancers [5,6,7], thus, further demonstrating the benefits of CT in pulmonary cancer diagnostics.

The criteria, to discern between benign and malignant pulmonary nodules in CT chest scans, are based mostly on morphological features such as size, shape, and growth [7,8,9,10], with nodule size and growth being the most important predictors of malignancy [9,11,12]. Cohering to RECIST (Response Evaluation Criteria in Solid Tumors), a nodule growth of more than 25% is labeled as a progressive disease [11]. Historically, diameter measurements have been the gold standard for estimating the size of pulmonary lesions [9,10,12]. It may be argued that, due to the high susceptibility of false-positive findings pertaining to this method [8,9], measuring volume and volume-doubling time in terms of nodule size and growth should be favored. As explained by de Hoop et al.: “Volumetric measurements are usually based on segmentation of nodules on thin section CT data sets and an algorithm that translates the segmented voxels into a nodule volume” [13]. Thanks to the emergence of semi-automated nodule volumetry software, volume measurements account for higher reproducibility and accuracy compared to manual diameter measurements [7,9,10,11,12,13].

Nonetheless, some of the challenges semi-automated software programs face are segmentation errors that may add to interexamination variability, and the variance of volumetry measurements between different semi-automated software packages [13]. Deficits in detecting “ground-glass” nodules and nodules located near pulmonary vessels and septa expose the limits of present, commercially available segmentation algorithms [11]. Furthermore, it is unclear if semi-automated volumetry measurements should be recommended over semi-automated diameter measurements since their performance may vary between different software packages [9]. Besides nodule segmentation software, other factors such as scan protocol, reconstruction algorithm, and nodule attenuation can inadvertently influence nodule size calculations as well [7,14].

Patients with pulmonary nodules which cannot be discriminated into benign or malignant lesions usually undergo repeated CT scan follow-ups. This can cause an unnecessary accumulation of radiation within potentially healthy patients [7,8,10]. To combat this problem, various dose-reduction strategies have been developed such as automatic tube current optimization, attenuation-based tube voltage selection, and noise reduction filters [6,7,8,10]. A promising avenue in this field is the use of iterative reconstruction combined with ultra-low-dose CT which could reduce radiation dose to that of a chest X-ray while simultaneously retaining measurement accuracy comparable to those found in standard-dose and low-dose CT [7,10]. In an article published by Schwyzer et al., the authors claim that dose reduction and iterative reconstruction settings affect the performance of automated nodule detection software by discovering fewer false-positive observations with iterative reconstruction set to a lower image noise output compared to settings generating higher image noise levels [14].

Still, despite these technological advancements, the interobserver variability of detecting and classifying pulmonary nodules remains high [6,15]. Moreover, inter-scanner variability by different CT manufactures may account for some of the variability found in CT images and should be considered in future examinations [16]. In an effort to standardize observer training and subsequently secure quality levels, the European Society of Thoracic Imaging (ESTI) has proposed the adherence to recommended technical standards for lung cancer CT screenings [17].

Considering the multitude of factors that can impact the interpretation of pulmonary nodule sightings on CT images, we must analyze the compounding effects of adjustable components, such as radiation dosage and iterative reconstruction, on measurement accuracy. With this study, it is our aim to elucidate and compare the precision and variability of different pulmonary nodule screening protocols with varying radiation dose levels and of disparate CT scanners from multiple manufacturers by semi-automatically measuring parameters such as nodule size, volume, and density in an anthropomorphic chest phantom with embedded lung nodules.

## 2. Materials and Methods

### 2.1. Chest Phantom

**Figure 1:**
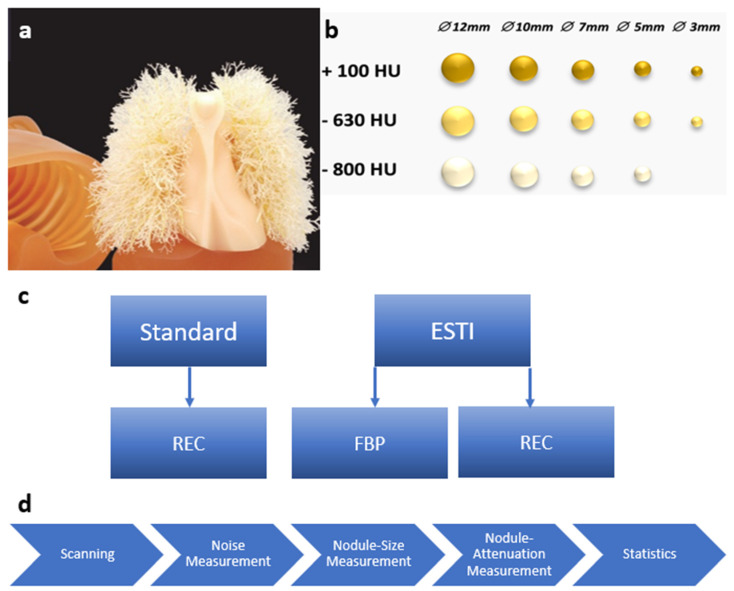
(**a**) Photo of the anthropomorphic chest phantom (Lungman Phantom, Kyoto Kagaku Co. LTD, https://www.kyotokagaku.com/en/products_data/ph-1_01/), access date: 7 February 2023. (**b**) Schematic overview of the lung nodules which were randomly placed in the phantom. (**c**) Scheme of scanning protocols with respective reconstructions. (**d**) Flow-chart of data processing and evaluation. Hounsfield units (HU). Filtered back Projection (FBP). Vendor-specific advanced reconstruction algorithm (REC). European Society of Thoracic Imaging (ESTI).

An anthropomorphic chest phantom (Lungman Phantom, Kyoto Kagaku Co. LTD, Kyoto, Japan) was used as an accurate anatomical model of a male human chest with arms in an abducted position. Phantom specifications are as follows: phantom size, 43 cm (width) × 20 cm (depth) × 46 cm (height); chest girth, 94 cm; phantom weight 18 kg. Its internal structures include mediastinum, bronchi, pulmonary vessels, and synthetic bones that have X-ray attenuation characteristics matching human tissues. Fourteen spherical pulmonary nodules (size ranging from 3 mm to 12 mm with Hounsfield units (HU) of −800 HU, −630 HU and +100 HU) were randomly placed throughout the lung (Figure 1).

### 2.2. CT Scanning Protocols

Images were acquired at five different CT scanners from four different manufactures (Naeotom Alpha and SOMATOM Edge Plus, Siemens Healthineers, Germany; Revolution GE Healthcare, United States; Aquillion One, Toshiba, Japan; and Spectral CT 7500, Philips, The Netherlands). Two different protocols were used for image acquisition: (1) the institution’s standard nodule protocol normally used on the respective scanner, (2) a protocol adapted according to the Lung Cancer Screening project of the ESTI [17]. While the standard protocol was reconstructed with an institution-specific, commonly used iterative reconstruction algorithm (REC), the ESTI protocols were reconstructed with filtered back projection (FBP) and REC, resulting in three different image sets per CT scanner. Detailed information on image acquisition protocols can be found in Table 1.

### 2.3. Image Noise Evaluation

Image noise was measured manually by one blinded reader by placing a circular region of interest (ROI) at three different levels of the trachea (carina, middle third and upper third of the trachea) as well as in the left and right main bronchus. Mean image noise was defined as the average of the standard deviation of the attenuation in the five consecutive ROI measurements [18].

### 2.4. Pulmonary Nodule Evaluation

#### 2.4.1. Nodule Density

Nodule density was measured manually by one blinded reader by placing a circular region of interest (ROI) in each nodule. Absolute CT attenuation for each nodule was recorded. To improve legibility, the phantom’s nodule densities and their corresponding measurements have been denominated and categorized as “solid” (+100 HU), “ground-glass 1” (−630 HU, GG1) or “ground-glass 2” (−800 HU, GG2).

#### 2.4.2. Nodule Size

One blinded reader performed semi-automated size measurements using a commercially available software package (MM Oncology, syngo.via, Siemens Healthineers, Forchheim, Germany) by placing a seed point in the nodule center to initiate semi-automatic nodule segmentation. Subsequently, the reader could evaluate segmentation and adjust the nodule segmentation manually if needed. Nodule volume and nodule diameter for each individual nodule were calculated automatically by the software and were recorded for each protocol and reconstruction.

### 2.5. Statistical Analysis

Calculated radiation dosages and background image noise were collated in search of significant differences among our selected scanning protocols by appropriately using two tailed *p*-tests and one-way ANOVAs. Information on the genuine size and density of our nodules was provided by the phantom’s manufacturer and served as a reference standard during size and density measurements. Measured (Vm) and calculated values (Vc) were used to compute the percentage errors of nodule volume and diameter measurements. According to Eberhard et al. [10], the absolute percentage error (APE) was calculated as 100 × (|(Vm − Vc)/Vc|) to indicate error margins and accuracy of nodule volumetry. Furthermore, a paired t-test was used to determine the differences in APE between nodule measurements (diameter vs. volume). In order to compare the attenuation accuracy of our scanning protocols among each other and with the actual nodule densities inside the phantom, we utilized ANOVA tests and determined *p*-values regarding the significance of any differences. To assess differences in nodule diameter, volume, and density among our protocols, we applied the proportional difference (PD) metric described by Bland and Altman as recommended [19]. This metric describes the PD of each measured nodule volume within the different protocols and is calculated as follows: 100 × (VPE − VPS)/(VPE + VPS). Mean differences assessed through Tukey tests are expressed as the mean and the 95% confidence interval (95% CI). A two-sided *p*-value below 0.05 (*p* < 0.05) was considered to indicate statistical significance. Statistical analysis has been performed using SPSS (Statistics software version 25, IBM, Armonk, NY, USA) and GraphPad Prism 9.0 (GraphPad Software Inc., San Diego, CA, USA). Continuous variables were expressed as mean +/− standard deviation (SD) while categorical variables were expressed as frequencies or percentages.

## 3. Results

### 3.1. Dose and Noise Evaluation

**Figure 2:**
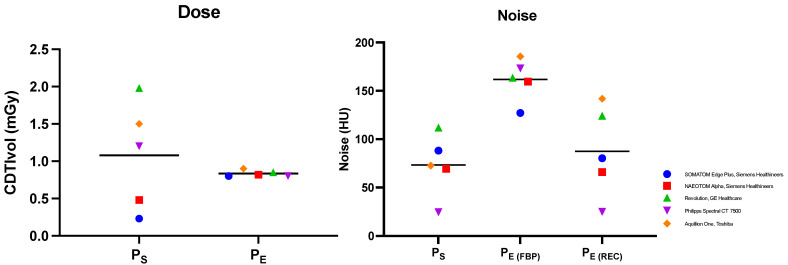
Comparison of radiation dose and image noise exposure. Observed mean dose (CDTIvol) and image noise (HU, Hounsfield Units) comparison between institute-specific standard low-dose protocols (P_S_) and ESTI protocols (P_E_) reconstructed with filtered back projection (P_E(FBP)_) as well as with iterative reconstruction (P_E(REC)_). The colors and shapes designate the CT scanner’s model and manufacturer.

When comparing radiation dosages between the standard protocols (P_S_) and ESTI protocols (P_E_), there was no significant difference (*p* = 0.48) to be found, with CDTIvol means being 1.1 ± 0.72 mGy for Ps and 0.8 ± 0.04 mGy for P_E_, respectively. However, the variance in dosage between the different institute-specific Ps was substantially higher than in P_E_ as implied by the visual below (Figure 2). There was significantly less background image noise in P_S_ and P_E(REC)_ than in the ESTI protocols reconstructed with FBP (P_E(FBP)_) (73.4 ± 29.98 HU and 87.5 ± 63.8 HU, respectively; vs. 161.8 ± 42.9 HU, *p* < 0.001). Yet, no significant difference was found between P_S_ and ESTI protocols with a conventional iterative reconstruction algorithm (P_E(REC)_) (73.4 ± 29.98 HU vs. 87.5 ± 63.8 HU, *p* > 0.49).

### 3.2. Nodule Size Evaluation

**Figure 3:**
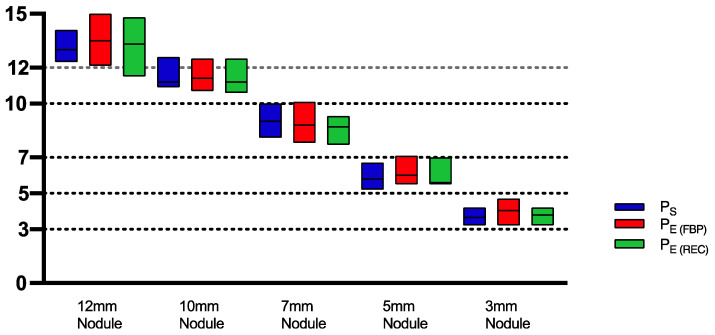
Box-plot diagram of mean ± SD diameter measurements of various nodules sizes (12, 10, 7, 5, 3 mm) among all protocols for all scanners showing nodule size overestimation. P_S_ = Standard Protocol, P_E(FBP)_ = ESTI Protocol with filtered back projection reconstruction algorithm applied, P_E(REC)_ = ESTI Protocol with conventional reconstruction.

**Figure 4:**
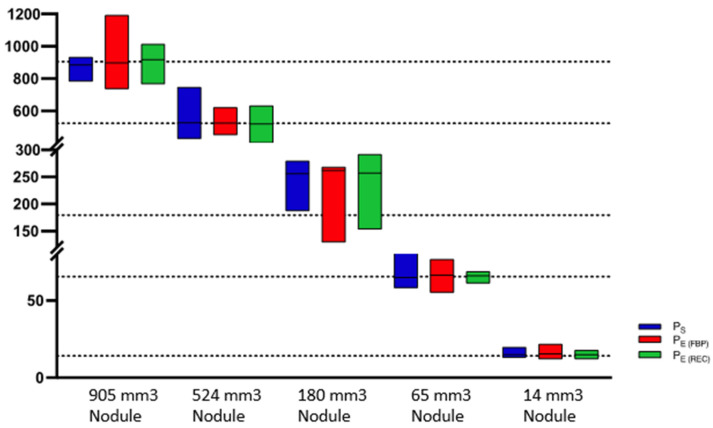
Box-plot diagram of mean ± SD volume measurements of various nodules sizes (905, 524, 180, 65, 14 mm^2^) among all protocols for all scanners included. P_S_ = Standard Protocol, P_E(FBP)_ = ESTI Protocol with filtered back projection reconstruction algorithm applied, P_E(REC)_ = ESTI Protocol with conventional reconstruction.

Irrespective of the protocol, reconstruction or scanner used, we have observed a systemic overestimation of nodule size in the evaluation of the nodule diameter as well as nodule volume (Figure 3 and Figure 4).

#### 3.2.1. Absolute Percentage Errors of Volume and Diameter Metrics

**Figure 5:**
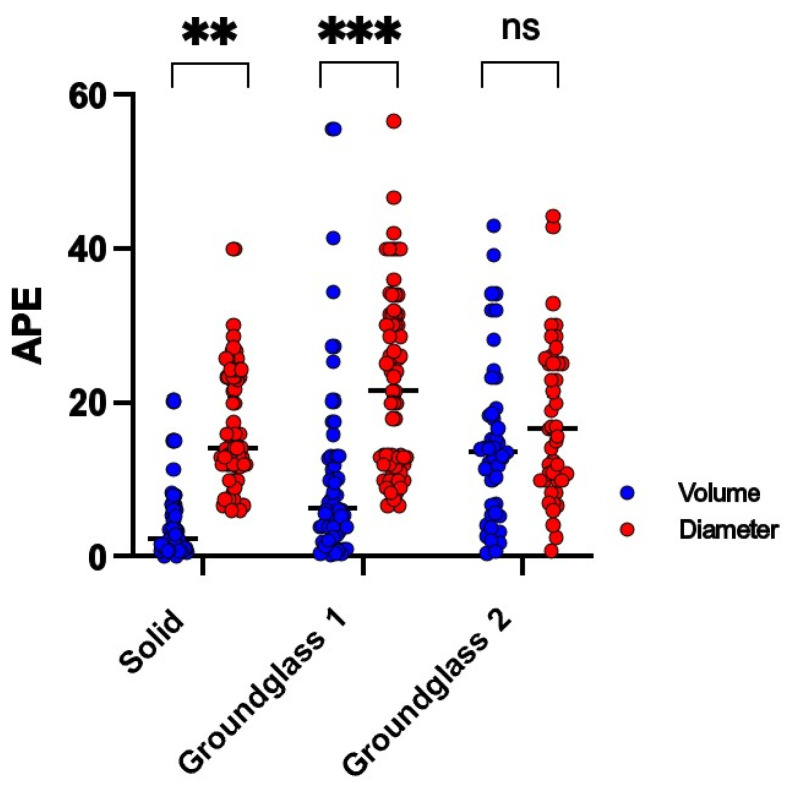
Absolute percentage errors (APEs) of quantified nodule volume and diameter. Nodules were separated and categorized according to density as solid, ground-glass 1 or ground-glass 2. The asterisk signifies the level of significance in APE difference between volume (blue) and diameter (red) measurements (**: *p* < 0.001; ***: *p* < 0.0001), whereas no significant difference (*p* > 0.05) is labeled as “ns”. Every dot represents the APE of a single measurement in its respective category.

After analyzing the APE between volume and diameter measurements amidst the different types of nodules across every CT model and protocol, there was a highly significant mean APE difference noticeable between measurements of volume and diameter within solid (4.2% ± 4.8% vs. 16.5% ± 7.8%, *p* < 0.001) and GG1 nodules (10.5% ± 12.1% vs. 22.2% ± 11.7%, *p* < 0.0001). Both these groups present a tendency of lower APE when measuring nodule volume in contrast to nodule diameter. The APE values were comparable between volume and diameter measurements among GG2 nodules (14.8% ± 10.7% vs. 17.7% ± 10.1%, *p* = 0.20) (Figure 5).

#### 3.2.2. Differences in Determined Nodule Size between Standard and ESTI Protocols

**Figure 6:**
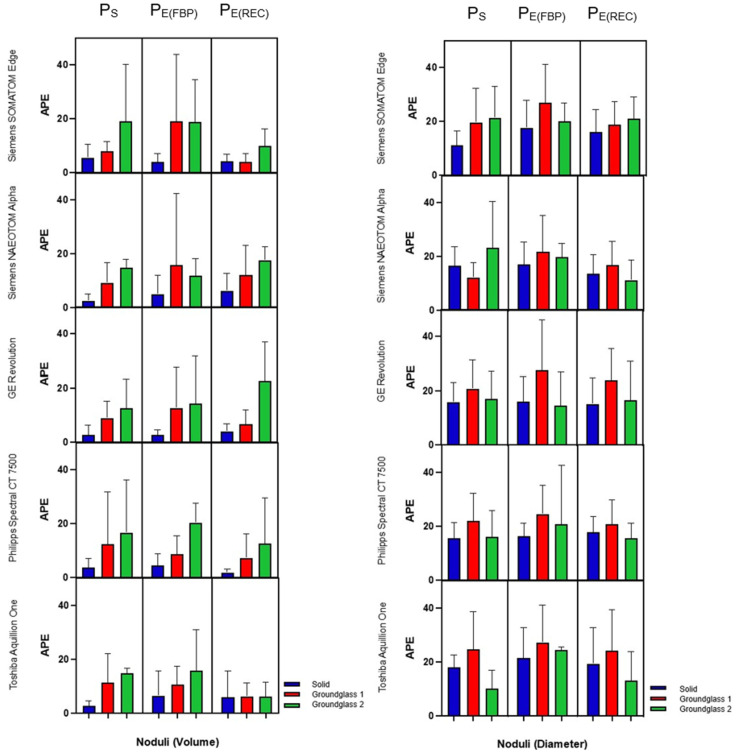
Box-plot diagram of absolute percentage errors (APEs) among different protocols and their respective computed tomography (CT) scanner. The colors indicate solid (blue), the ground-glass 1 (red) and ground-glass 2 (green) nodules. Measured nodule volumes are represented on the left and their corresponding diameters on the right. Institute-specific standard low-dose protocols (P_S_) and ESTI protocols (P_E_) reconstructed with filtered back projection (P_E(FBP)_) as well as with iterative reconstruction (P_E(REC)_) were compared.

The mean differences observed among P_S_, P_E(FBP)_ and P_E(REC)_ for volumetric as well as for diametric measurements were insignificant (*p* = 0.12–0.99), as portrayed in Table 2. When comparing the APE for volume measurements across all nodule sizes, solid nodules have shown the lowest APE, with mean APEs of 3.2% ± 3.35% (P_S_), 4.1% ± 5.1% (P_E(FBP)_) and 4.9% ± 5.0% (P_E(REC)_). The highest mean APEs for volume measurements were found in GG2 nodules with 14.78% ± 10.1% (P_S_), 14.97% ± 12.9% (P_E(FBP)_) and 15.79% ± 10.9% (P_E(REC)_). The APEs for diameter measurements in P_S_ and P_E(FBP)_ were lowest in solid nodules, with means of 15.3% ± 6.0% and 17.7% ± 8.5%, respectively. In P_E(REC)_, however, GG2 nodules had the lowest mean APE of around 15.5% ± 9.0%. The highest mean APEs of diameter measurements regarding all protocols were found within GG1 nodules with 20.2% ± 10.9 (P_S_), 25.7% ± 13.3% (P_E(FBP)_) and 20.9% ± 10.4% (P_E(REC)_) (Figure 6).

### 3.3. CT Attenuation Evaluation

#### 3.3.1. Comparison between Protocol Determined CT Attenuation and Factual Nodule Density

**Figure 7:**
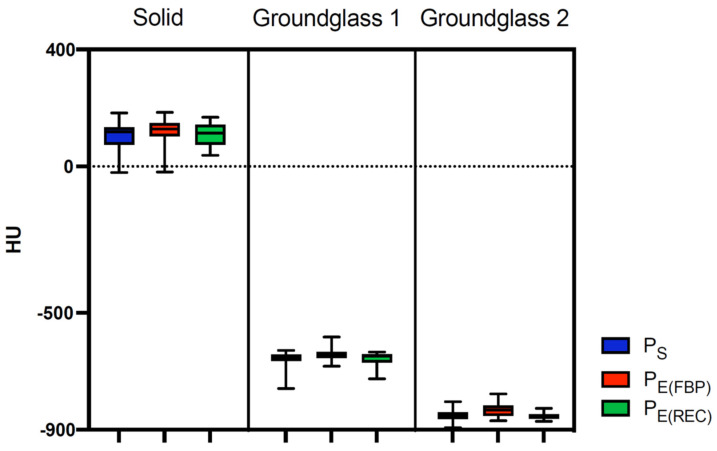
Box-plot diagram of measured CT attenuation according to scanning protocol and nodule density. The graph visualizes the spread of observed attenuation (HU, Hounsfield Units) among different factual nodule densities and scanning protocols. Colors represent the utilized scan protocol. Institute-specific standard low-dose protocols (P_S_), ESTI protocols (P_E_) reconstructed with filtered back projection (P_E(FBP)_) as well as iterative reconstruction (P_E(REC)_) were compared.

After examination, we perceived no significant divergence (*p* = 0.06–0.8) between the CT-expressed attenuation and the reference density of the phantom’s solid nodules, as demonstrated in Table 3 and Figure 7. The highest mean difference in this group was noted in P_E(FBP)_ (−20.51 HU) and lowest in P_S_ (−6.68 HU). In contrast, GG1 and GG2 exhibited highly significant disparities (*p* < 0.0001–0.03) between observed CT attenuation and density reference values across all protocols. The mean differences were most prominent with GG2 nodules in P_E(REC)_ showing the largest (52.4 HU), and the lowest (33.35 HU) in P_E(FBP)_, divergence. Last, while assessing our GG1 nodules, we discerned the greatest mean difference in P_S_ (31.6 HU) and the smallest in P_E(FBP)_ (12.36 HU).

#### 3.3.2. Comparison of CT Attenuation among Different Scan Protocols

With regard to CT attenuation, dissimilarities between scan protocols were consistently insignificant (*p* = 0.07–0.97) with few exceptions (Table 4). In particular, significant discrepancies were particularly observed between P_E(FBP)_ and P_E(REC)_ in both classes of ground-glass nodules, with P_E(FBP)_-based CT attenuation being closer to the targeted value of the nodule’s factual density. This was also true when comparing P_S_ with P_E(FBP)_ in GG2 nodules. In each respective nodule density group, the smallest mean difference was seen between P_S_ and P_E(REC)_ (solid: −3.724 HU; GG1: −6.28 HU; GG2: 2.6 HU). The highest mean differences were identified between P_S_ and P_E(FBP)_ in solid (−13.83 HU) and GG1 (−19.24 HU) nodules, whereas in GG2 nodules, it was found to be between P_E(FBP)_ and P_E(REC)_ (19.05 HU).

## 4. Discussion

In this study, we compared different pulmonary nodule screening protocols and disparate CT scanners from multiple manufacturers by semi-automatically evaluating nodules in an anthropomorphic chest phantom. We found that, when considering radiation dose, image noise and comparability of nodule size and density measurements, P_E(REC)_ is more favorable over P_S_.

Although the mean radiation doses between P_S_ and P_E_ were similar, there was substantial variance in radiation dose among P_S_. This might be due to individual preferences set by the different institutions and recommendations for pulmonary nodule imaging by a CT-providing vendor. Yet, our results imply that by implementing P_E_, we can standardize radiation dose and decrease variability between different institutions and CT scanners.

Specifically, ESTI guidelines suggest a radiation dose of 0.8 mGy for patients between 50 and 80 kg and is applicable to any multidetector CT with 32 rows or more [17]. Any scanning protocol with a higher dosage setting could, therefore, potentially benefit from these guidelines by lowering dose levels and consequently reducing the risk of radiation-induced carcinogenesis. 

As Goldman [20] explains, image noise is one of four basic factors (the others being spatial resolution, image contrast, and artifacts) which influence image quality in CT scans and if reduced, may improve the visibility of low-contrast structures. Currently, in low-dose CT iterative reconstruction, algorithms are used to decrease image noise in order to obtain diagnostic images despite the low dose [21,22,23]. As expected, our study image noise was lower in images reconstructed with iterative reconstruction algorithms than in those reconstructed with FBP. Between P_S_ and P_E(REC)_, there was no statistically significant difference in image noise. 

Our results exalt the advantage of choosing volumetric over diametric measuring tools in terms of accurately estimating the size of solid nodules. This coincides with claims and recommendations made by various publications [7,9,10,11,12,13,24].

However, not all of our ground-glass nodules presented a significant improvement in favor of volume measurements. The insufficiencies of the utilized computer-aided detection software to correctly recognize the borders of low-density ground-glass nodules subsequently required the observer to redraw the nodule’s circumference by hand and thus inherently added inaccuracy and variability to our measurements. The limits of detecting small ground-glass nodules with semi-automated segmentation programs are described in the literature [11,25,26,27].

Another explanation for the relatively large APEs within our data can be contributed to the erroneous segmentation of smaller nodules which create greater variability than equally great segmentation errors of larger nodules [7,10].

Although no statistically significant difference in precision could be asserted between our selected protocols, we did notice a trend of slightly smaller APEs within protocols that applied iterative reconstructions algorithms. As previously discussed, FBP algorithms result in higher ratios of image noise, and therefore, reduced image quality when compared to modern iterative reconstruction programs which can potentially hamper the performance of semiquantitative measurements [21,22,23]. Because of this, an argument can be made to prefer P_E(REC)_ over P_S_, which irradiates higher radiation dosages, but does not offer more accuracy. 

An evaluation of absolute measurement errors showed an overestimation of nodule diameter and nodule volume irrespective of the protocol, reconstruction or scanner used. Since this seems to be a systemic error, it should play a minor role in follow-up imaging, where the main focus is to detect size differences, i.e., the detection of nodule growth. 

The absence of any significant deviation between protocol-based CT attenuation and the solid nodules’ true density values exemplifies the precision of our acquired scanning protocols. Scanning protocols which employed an iterative reconstruction program especially seemed to be more on-target than those that did not. Albeit so, the comparative differences in the accurate attenuation of solid nodule densities between our protocols were insignificant with the lowest mean difference between P_S_ and P_E(REC)_, again declaring the proposition that P_E_ may replace current P_S_ with higher radiation dosages without suffering much loss in image quality. A phantom study conducted by Kim et al. [28] reached similar results, proclaiming that there was no significant difference in CT attenuation between FBP and iterative model reconstruction in solid nodules. This is in-line with our study, where there was no statistically significant difference in measured CT attenuation for solid nodules among images reconstructed with FBP or iterative reconstruction.

Intriguingly, our examination of ground-glass nodules indicates that P_E(FBP)_ was more exact in rendering the actual phantom nodule’s density than protocols reconstructed with iterative reconstruction. Nevertheless, images generated with iterative reconstruction had more robust and homogenous values than P_E(FBP)_ across different scan protocols and CT machines from different vendors. In clinical practice, measuring the change in nodule’s CT attenuation over time can help assess its malignancy, and an increase in CT attenuation in ground-glass nodules coincides with a higher chance of malignant behavior [29,30,31,32,33]. Therefore, protocols that express less variability between different follow-up scans, CT scanners, and iterative reconstruction algorithms may lead to fewer misinterpretations of aberrant nodule CT attenuation reported in CT follow-ups and should be favored over protocols with higher variance.

Besides ESTI, the American College of Radiology (ACR) also published recommendations for LCS protocols on their website [34]. All these recommendations have the aim to homogenize screening protocols throughout different institutions in order to (a) keep radiation exposure to patients low and (b) to make scans performed at different institutions more comparable between each other.

Our study has the following limitations: First, in light of the circumstance that some of the semi-automated measurements were corrected manually, we must assume a certain amount of inadvertent variability and human error in our results. Intra- as well as interobserver variability of manual nodule size evaluations can be considerable as described by Bogot et al. [32]. Because the chosen nodule characteristics have been measured only once by a single blinded reader, we cannot attest to the scale of intra- or interobserver variance of our examined protocols. 

Second, pulmonary vessels, septa and other anatomical structures adjacent to nodules can confound end results [11,26] by miscalculating the edge of a pulmonary nodule through semi-automated segmentation programs or by unintentionally, manually including them into the ROI while assessing CT attenuation. 

Third, needless to say, our study offers insight only into a small window of commercially available CT scanners, reconstruction algorithms and scanning protocols. By increasing our sample size, we could generate results based on sounder statistical footing [33].

Fourth, not all scanners offered the exact value for all of the by ESTI-suggested scanning parameters. Where exact parameters were not available, the closest option was chosen.

Fifth, inhomogeneity in nodule CT attenuation is an important finding in daily clinical practice since solid parts in otherwise ground-glass nodules are an indicator of malignancy [35]. We were not able to investigate nodule inhomogeneity in our study since our phantom was only equipped by the vendor with solid and pure ground-glass nodules. 

## 5. Conclusions

Taking into account radiation dose, image noise and comparability of nodule size and density measurements, the ESTI-recommended screening protocol with use of iterative reconstruction has the most favorable outcome. To answer the question posed in the title, we recommend favoring volumetric over diametric measurements, specifically when evaluating the size and growth of solid pulmonary nodules. 

Regardless, challenges persist with the current semi-automated detection of pulmonary ground-glass nodules. Segmentation errors force manual correction, adding the human factor with all its variability and inaccuracies. In the worst case, nodule detection can be overlooked in pulmonary CT screenings. It is our belief that the developing improvements in semi-automated segmentation programs will address these limitations.

## Figures and Tables

**Table 1:** Scanning Protocols. The tube current of the NAEOTOM scanner was modulated by adjusting the system’s image quality (IQ) setting. The standard protocol of GE’s Revolution CT scanner used a rotational automated exposure control program named SmartmA. The standard protocol used by Toshiba’s Aquillion One and an automated exposure control program designated by the letters “A” and “R”. CTDI: Computed tomography dose index; DLP: Dose length product; 100 sn: 100 kV with tin filtration; ADMIRE: Advanced Modeled Iterative Reconstruction; QIR: Quantum Iterative Reconstruction; ASiR: Adaptive Statistical Iterative Reconstruction; AIDR: Adaptive Iterative Dose Reduction; IMR: Iterative Model Reconstruction. Filtered Back Projection (FBP).

CT Scanner		SOMATOM Edge Plus, Siemens Healthineers			NAEOTOM Alpha, Siemens Healthineers			Revolution, GE Healthcare			Aquillion One, Toshiba			Spectral CT 7500, Philips		
Scan Protocol		Standard	ESTI		Standard	ESTI		Standard	ESTI		Standard	ESTI		Standard	ESTI	
Dose	CTDI (mGy)	0.23	0.8		0.48	0.82		1.98	0.85		1.5	0.9		1.2	0.8	
	DLP (mGy*cm)	8.25	29		16.3	28.2		78.24	32.61		54.8	32.3		52.1	36.7	
Tube voltage (kV)		100 sn	100 sn		120	100 sn		120	100		A 80	100		120	100	
Tube current (mAs)		100	310		IQ level 5	IQ level 20		SmartmA 10–400	40		R 165	35		15 (10–20)	17 (11–24)	
Pitch		1.2	1.2		0.85	1		0.992	0.992		0.5 × 80	0.5 × 80		1.15	1.15	
Rotation time (s)		0.5	0.5		0.5	0.5		0.5	0.5		0.35	0.35		0.33	0.33	
Slice Thickness (mm)		1.5	1		1.5	1		0.625	0.625		1	1		1	1	
Increment (mm)		1	0.7		1	0.7		0.625	0.7		0.8	0.7		0.5	0.7	
Reconstruction algorithm		ADMIRE 3	ADMIRE 3	FBP	QIR 3	QIR 3	FBP	ASiR-V 50%	ASiR-V 50%	FBP	AIDR 3D standard	AIDR 3D standard	FBP	IMR level 1	IMR level 1	FBP

**Table 2:** Comparative overview of measured nodule size differences among distinct protocols. The table displays and compares the metric differences between institute-specific standard low-dose protocols (P_S_) and ESTI protocols (P_E_) reconstructed with filtered back projection (P_E(FBP)_) as well as with iterative reconstruction (P_E(REC)_). CI: Confidence Interval.

Metric	Protocol	Mean 1/Mean 2	95% CI of Difference	*p*-Value
Volume	P_S_ vs. P_E(FBP)_	9.098/10.87	−6.549 to 3.000	0.6543
	P_S_ vs. P_E(REC)_	9.098/7.990	−3.666 to 5.882	0.8472
	P_E(FBP)_ vs. P_E(REC)_	10.87/7.990	−1.892 to 7.657	0.3290
Diameter	P_S_ vs. P_E(FBP)_	17.71/21.29	−7.861 to 0.7025	0.1213
	P_S_ vs. P_E(REC)_	17.71/17.90	−4.432 to 4.066	0.9943
	P_E(FBP)_ vs. P_E(REC)_	21.29/17.90	−0.8522 to 7.645	0.1448

**Table 3:** Mean difference of attenuated and objective nodule densities. The table above displays the mean, mean difference and *p*-value of each protocol compared to the genuine nodule density (HU, Hounsfield Units). Institute-specific standard low-dose protocols (P_S_) and ESTI protocols (P_E_) reconstructed with filtered back projection (P_E(FBP)_) as well as with iterative reconstruction (P_E(REC)_) were compared.

Nodule Density	Protocol	Mean [HU]	Mean Difference [HU]	*p*-Value
Solid (100 HU)	P_s_	106.7 ± 49	−6.68	0.8357
	P_E(FBP)_	120.5 ± 42.1	−20.51	0.0578
	P_E(REC)_	110.4 ± 39.3	−10.4	0.4234
Ground-glass 1 (−630 HU)	P_s_	−661.6 ± 30	31.6	<0.0001
	P_E(FBP)_	−642.4 ± 22.3	12.36	0.0282
	P_E(REC)_	−655.3 ± 22.1	25.32	<0.0001
Ground-glass 2 (−800 HU)	P_s_	−849.8 ± 20.1	49.8	<0.0001
	P_E(FBP)_	−833.4 ± 22.4	33.35	<0.0001
	P_E(REC)_	−852.4 ± 11.2	52.4	<0.0001

**Table 4:** Differences in observed attenuation amidst different scan protocols. The table above exhibits and compares the difference of measured CT attenuation (HU, Hounsfield Units) among the selected scan protocols. Institute-specific standard low-dose protocols (P_S_) and ESTI protocols (P_E_) reconstructed with filtered back projection (P_E(FBP)_) as well as with iterative reconstruction (P_E(REC)_) were compared.

Nodule Density	Protocol	Mean [HU]		Mean Difference [HU]	*p*-Value
Solid (100 HU)	P_s_ vs. P_E(FBP)_	106.7	120.5	−13.83	0.2355
	P_s_ vs. P_E(REC)_	106.7	110.4	−3.724	0.9738
	P_E(FBP)_ vs. P_E(REC)_	120.5	110.4	10.1	0.5162
Ground-glass 1 (−630 HU)	P_s_ vs. P_E(FBP)_	−661.6	−642.4	−19.24	0.0668
	P_s_ vs. P_E(REC)_	−661.6	−655.3	−6.28	0.5699
	P_E(FBP)_ vs. P_E(REC)_	−642.4	−655.3	12.96	0.0194
Ground-glass 2 (−800 HU)	P_s_ vs. P_E(FBP)_	−849.8	−833.4	−16.45	0.0007
	P_s_ vs. P_E(REC)_	−849.8	−852.4	2.6	0.8715
	P_E(FBP)_ vs. P_E(REC)_	−833.4	−852.4	19.05	0.0003

## Data Availability

Data are available upon request.
